# Fluctuation and change of serum urate levels and flares in gout: results from the NOR-Gout study

**DOI:** 10.1007/s10067-022-06416-4

**Published:** 2022-11-01

**Authors:** T. Uhlig, L. F. Karoliussen, J. Sexton, T. K. Kvien, E. A. Haavardsholm, F. Perez-Ruiz, H. B. Hammer

**Affiliations:** 1 Center for Treatment of Rheumatic and Musculoskeletal Diseases (REMEDY), Diakonhjemmet Hospital, Box 23, Vinderen, N-0319 Oslo, Norway; 2 Faculty of Medicine, University of Oslo, Oslo, Norway; 3 Division of Rheumatology, OSI EE-Cruces, Cruces University Hospital, Osakidetza, Barakaldo, Spain; 4 Biocruces-Bizkaia Health Research Institute, Barakaldo, Spain; 5 Medicine Department, Medicine School, University of the Basque Country, Leioa, Spain

**Keywords:** Flare, Fluctuation, Gout, Serum urate, Treat to target, Urate-lowering treatment

## Abstract

A gout attack may evolve after a purine-rich diet or alcohol and after starting urate-lowering therapy (ULT). The relationships between fluctuation and change in serum urate (SU) with the occurrence of flares were investigated in this study. In the prospective NOR-Gout study, gout patients with increased SU and a recent flare were treated to target with ULT over 1 year, with follow-up at year 2 with SU and flare as outcomes. SU and flares were assessed at both monthly and 3-monthly intervals until target SU was reached. Fluctuation over periods and changes in SU between two time points were assessed and compared in patients with and without flares. At year 1, 186 patients completed follow-up (88.2%) and 173 (82.0%) at year 2. Mean age (SD) at baseline was 56.4 (13.7) years, disease duration was 7.8 (7.6) years, and 95.3% were men. The first-year SU fluctuation and change were related to flare occurrence during year 1 (both *p* < 0.05). High fluctuation with an absolute sum of all SU changes during the first 9 months was related to flares over 3-month periods (all *p* < 0.05), and high fluctuation during the first 3 months was related to flares in months 3–6 (*p* = 0.04). Monthly and high SU changes or again reaching higher SU levels > 360 µmol/l were not related to flares. Fluctuation and change in SU were related to flare occurrence during the first year of ULT, while changes between visits and reaching SU levels > 360 µmol/L were not related to flares.

**Table:** 

**Key Points**
• *Urate-lowering therapy seeks to achieve a treatment target and prevent gout flares, and changes in serum urate are related to gout flares.*
• *Fluctuation and changes in serum urate were associated with gout flares, suggesting that fluctuation in serum urate is unfavourable during gout treatment.*
• *During urate-lowering therapy in gout in clinical practice, fluctuation of serum urate, for example, due to lack of adherence, should be observed and avoided.*

## Introduction

In gout, a prevalent inflammatory joint disease [1–3], hyperuricaemia leads to the formation of monosodium urate (MSU) crystals, followed by flares of inflammation and pain. Urate-lowering therapy (ULT) seeks to reduce serum urate (SU) and prevent gout flares [4, 5]. Flares are frequent after the start of ULT, especially during the first 3–6 months [6, 7], and prophylactic flare treatment is therefore recommended [4].

A gout attack may evolve after a purine-rich diet or alcohol, triggered by a proinflammatory effect of temporary increased serum urate [8]. Flares have also been associated with decreases and fluctuations in urate levels in response to pegloticase treatment [9], a finding which supports the hypothesis that not momentary SU levels but rather fluctuations could initiate a flare. Fluctuation of SU is also observed after bariatric surgery with an immediate increase of SU and then a decline after weeks until years [10–12], and increased flares are observed postoperatively [10, 13–15]. Thus, gout flares could be triggered by fluctuation in SU over time, but evidence lacks, especially in routine ULT. We studied whether fluctuation and changes in SU during an intensive ULT approach were related to gout flares over two years.

## Materials and methods

### Study design and participants

NOR-Gout (Gout in Norway) is a prospective, observational single-centre study in a hospital-based rheumatology centre where patients satisfying ACR/EULAR classification criteria [16] and with crystal-proven gout were treated with ULT. They were included after a recent flare if SU > 360 μmol/L, ACTRN12618001372279 [17]. The study was approved by the regional ethics committee, included patient representatives in project planning, and was performed in accordance with the Declaration of Helsinki and Good Clinical Practice guidelines. All patients provided written informed consent. Diakonhjemmet Hospital sponsored the study.

After a baseline rheumatology outpatient visit at Diakonhjemmet Hospital, ULT was started with allopurinol 100 mg daily with 100 mg increments monthly until treatment target SU < 360 μmol/L (or < 300 μmol/L if clinical tophi were present) was reached, with the maintenance of this dose. Patients switched to febuxostat in case of intolerance or lack of efficacy. Compliance with medication was not specifically tested. Flare prophylaxis was at the discretion of the treating rheumatologist and included colchicine 0.5–1 mg daily for 3–6 months [17] in all but six patients who used low-dose prednisolone.

### Visits, outcome, and covariates

Study visits assessed SU and flare status [7] after 1 month, 2 months, 3 months, 4 months, 6 months, 9 months, and 12 months during year 1 independent of time of flare occurrence, and then after year 2, with additional monthly visits in year 1 if required unless target SU was met.

The primary outcomes were SU and flares after the first year. At every clinical visit, the patient self-reported gout flares since the last visit during a structured interview with a trained study nurse who recorded the flares. If in doubt, the patient and the study nurse discussed whether an experienced episode with pain or swelling was to be defined as a gout flare or not. At all visits, laboratory examinations included SU (µmol/L).

Fluctuation was used as a concept for accumulated positive and negative changes in SU, and was defined as the absolute sum of all observed SU changes between adjacent visits accumulated from baseline to time of observation, i.e. when flare occurrence was studied. SU change was defined as the difference between two visits, between 3-month time points or between years 1 and 2. SU fluctuation and change were compared in patients with and without flares during defined periods.

We also studied the effect of larger SU changes between two visits with thresholds > 30 µmol/L, > 60 µmol/L and > 90 µmol/L for flare occurrence. Finally, we investigated flares among patients who, during the study, had reached the treatment target of < 360 µmol/L. We compared whether a consecutive SU increase to levels > 360 µmol/L and > 400 µmol/L, where an increased risk of MSU crystal formation is anticipated, was associated with flares.

### Statistics

Descriptive measures for baseline demographics, SU levels and changes during the study and frequency of flares were explored with means with differences between groups using independent samples T-test and by the χ^2^ test or Fisher’s exact. Adjustments for multiple comparisons were not done. *P* < 0.05 was defined as statistically significant. Calculations were performed with IBM SPSS statistics (version 27).

## Results

### Patient characteristics and visits

**Fig. 1:**
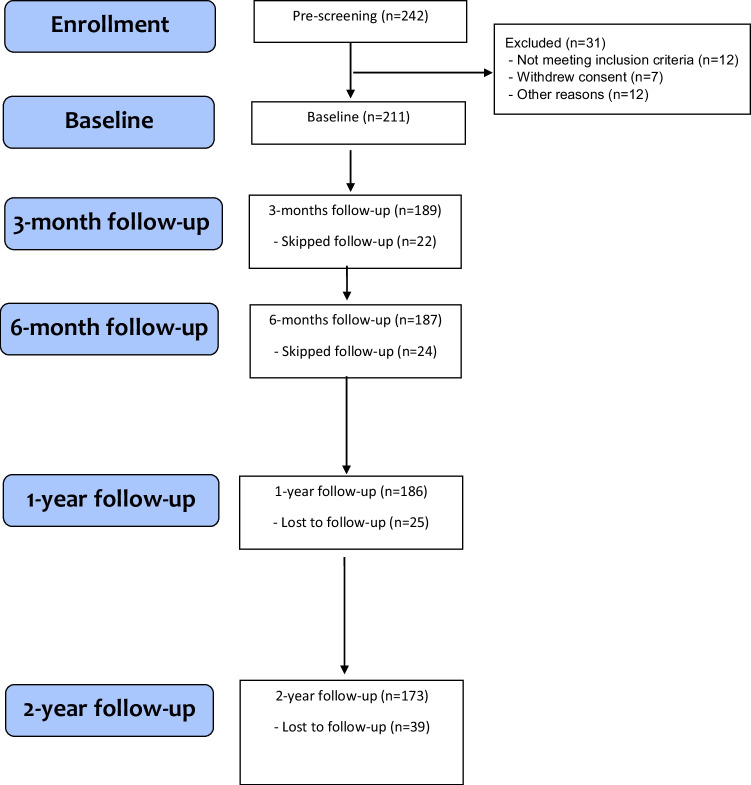
STROBE flow diagram of NOR-Gout participants with main examinations

Patient numbers (Fig. 1) at visits were 211 (baseline), 202 (month 1), 193 (month 2), 189 (month 3), 176 (month 4), 167 (month 9), 187 (month 6), 186 (month 12), 173 (24 months) and between 27 and 75 patients met for additional visits. Mean age (SD) at baseline was 56.4(13.7) years, disease duration 7.8(7.6) years, 95.3% were men, 16.6% had clinical tophi, and 73.4% had at least two flares in the year before inclusion.

### SU changes, fluctuations and flares over 2 years

Mean SU decreased during ULT from 500 (SD 77) to 311 (SD 49) µmol/L in year 1 and 325 (SD 70) µmol/L in year 2. For fluctuation, the mean sum of SU changes from baseline was 196 µmol/L (until 3 months), 274 µmol/L (6 months), 307 µmol/L (9 months), 366 µmol/L (12 months) and 412 µmol/L (until 24 months). A flare during the study was experienced by 80.6% (150/187) during year 1 and 26.0% (45/173) during year 2.

### Association between SU fluctuation and flare

**Fig. 2:**
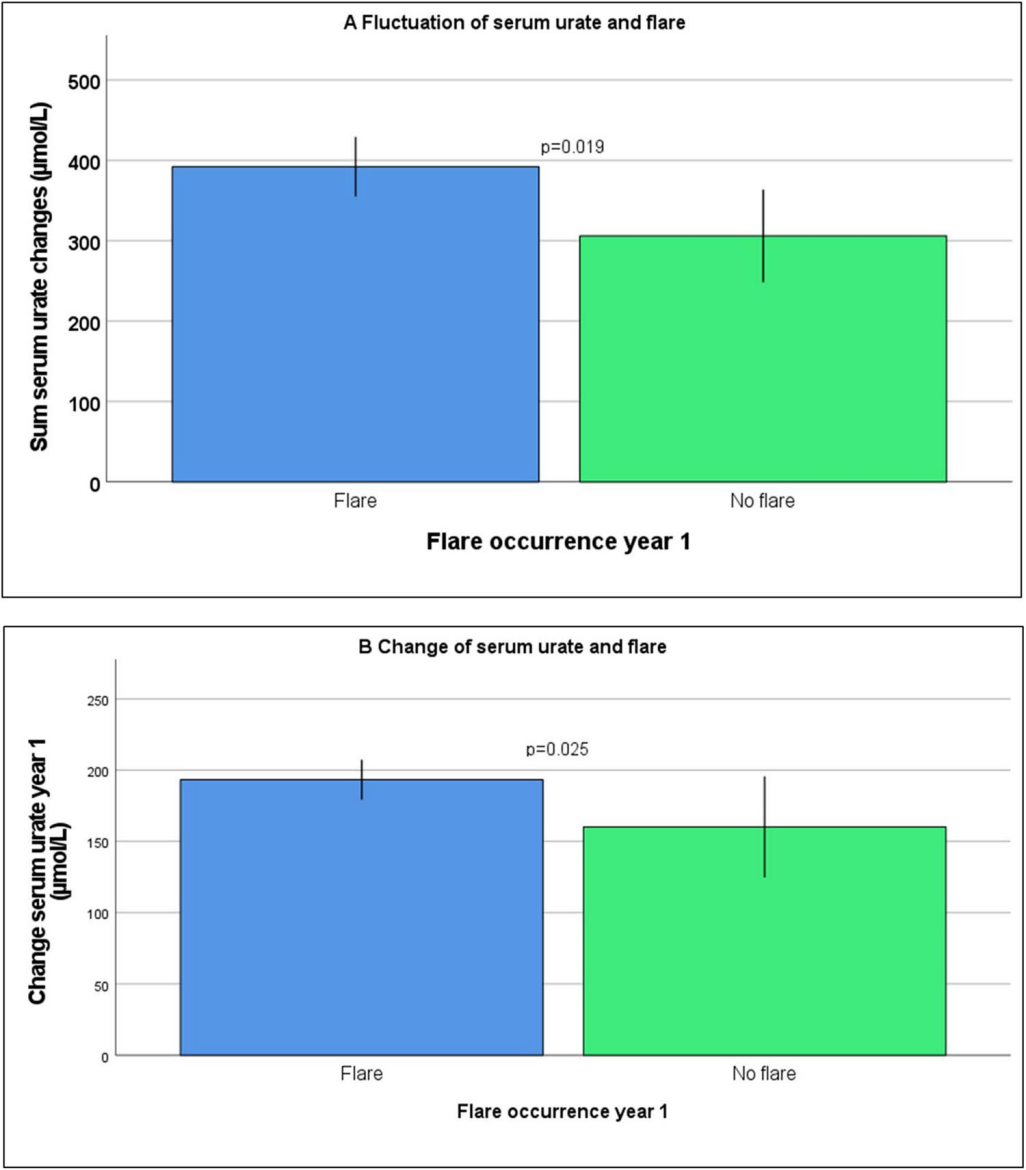
Fluctuation and serum urate change over 1 year (means with 95% confidence intervals)

During year 1, the sum of changes (392 vs. 306 µmol/L, *p* = 0.019) was higher in patients with vs. patients without a flare (Fig. 2).

SU fluctuation during the increasing observation period is related to flare occurrence in 3-month periods. Table 1 shows fluctuation for patients with and without such flares at the end of the observation period and in the subsequent 3-month period. SU fluctuation with sum of changes was higher in patients with flare vs. no flare during the first 3 months (*p* = 0.026), months 3–6 (*p* = 0.028) and months 6–9 (*p* = 0.029). Furthermore, fluctuation until month 3 related to subsequent flare during months 3–6 (*p* = 0.038).

**Table 1:** Fluctuation of serum urate (mean sum of absolute changes) and flare occurrence in last observed and in a subsequent 3-month period or year 2

Period (months)	All patients µmol/L (SD)	Flare status	Sum of SU changes and period for flare (months)				
			0–3	3–6	6–9	9–12	12–24
	*n* = 205		No. Flare + /Flare − 59/139	No. Flare + /Flare − 85/94	No. Flare + /Flare − 31/113	No. Flare + /Flare − 69/104	No. Flare + /Flare − 45/128
0–3	196 (93)	Flare +	203 (106)	210 (104)			
		Flare −	193 (87) *p = 0.026	186 (82) *p = 0.038			
0–6	274 (140)	Flare +		295 (142)	288 (167)		
		Flare −		257 (137) *p = 0.028	269 (12) *p* = 0.18		
0–9	307 (166)	Flare +			351 (221)	323 (178)	
		Flare −			291 (137) *p = 0.029	310 (163) *p* = 0.31	
0–12	366 (223)	Flare +				409 (251)	357 (221)
		Flare −				360 (208) *p* = 0.08	394 (236) *p* = 0.18
0–24	413 (247)	Flare +					411 (240)
		Flare −					449 (259) *p* = 0.19

^*^P < 0.05 for comparison Flare + versus Flare −

### Association between SU change and flare

During year 1, SU change was higher in patients with vs. without a flare (193 vs. 160, *p* = 0.025) (Fig. 2).

Monthly or 3-monthly SU changes were not related to flares (Table 2), neither for patients who exceeded threshold changes of 30 to 90 µmol/L.

**Table 2:** Comparison between patients with and without flares regarding monthly serum urate (SU) change exceeding thresholds

Month(s)		*N*	Mean SU change	*p*-value	SU change, > 30 µmol/L (%)	*p*-value, flare vs. no flare	SU change, > 60 µmol/L (%)	*p*-value, flare vs. no flare	SU change, > 90 µmol/L (%)	*p*-value, flare vs. no flare
0–1	Flare +	37	102	0.16	81.1	0.64	62.2	0.76	48.6	0.41
	Flare −	165	82		77.6		64.8		41.2	
1–2	Flare +	25	25	0.06	48.0	0.30	32.0	0.31	24.0	0.89
	Flare −	166	47		49.0		42.8		25.3	
2–3	Flare +	28	27	0.40	46.4	0.86	28.6	0.94	10.7	0.47
	Flare −	150	30		47.7		29.3		16.0	
3–4	Flare +	41	3	0.28	29.3	0.35	14.6	0.32	25.0	0.93
	Flare −	129	11		37.2		21.7		75.0	
4–5	Flare +	14	24	0.24	50.0	0.90	28.6	0.97	7.1	0.28
	Flare −	52	35		48.1		30.8		19.2	
5–6	Flare +	21	23	0.034	38.1	0.12	23.8	0.22	14.3	0.13
	Flare −	49	− 7		20.4		12.2		4.1	
6–7	Flare +	14	42	0.08	57.1	0.09	35.7	0.22	21.4	0.14
	Flare −	41	13		31.7		19.5		7.3	
7–8	Flare +	6	33	0.15	50.0	0.51	33.3	0.07	0	0.64
	Flare −	28	− 4		35.7		7.1		3.6	
8–9	Flare +	4	− 18	0.30	25.0	0.63	25.0	0.77	0	0.34
	Flare −	32	5		37.5		18.8		18.8	
9–10	Flare +	13	56	0.19	61.5	0.16	38.5	0.60	38.5	0.26
	Flare −	36	30		38.9		30.6		22.2	
10–11	Flare +	4	− 38	0.14	25.0	0.56	0	0.29	0	0.59
	Flare −	22	12		13.6		22.7		13	
11–12	Flare +	8	6	0.46	25.0	0.61	0	0.71	0	ND
	Flare −	20	7		15.0		5.0		0	
Period										
0–3	Flare +	59	141	0.046	88.1	0.07	83.1	0.08	67.8	0.051
	Flare −	139	165		95.5		91.5		80.8	
3–6	Flare +	85	19	0.44	38.8	0.94	28.2	0.57	10.6	0.39
	Flare −	94	17		39.4		24.5		14.9	
6–9	Flare +	47	10	0.10	49.0	0.005	25.5	0.11	25.5	0.13
	Flare −	110	26		26.5		15.5		15.5	
9–12	Flare +	59	4	0.34	32.2	0.18	16.9	0.16	11.9	0.24
	Flare −	104	9		22.6		9.4		6.6	
12–24	Flare +	41	− 1	0.15	24.4	0.86	11.1	0.91	6.7	0.70
	Flare −	113	11		25.8		11.7		4.7	

*P*-value with independent *t*-test or chi-squared/Fisher exact test for comparisons flare versus no flare

*ND*, not determinable

SU changes over 3-month periods and possible associations to flare occurrence at the next subsequent visit are shown in Table 3, not demonstrating consistent differences between patients with and without flare.

**Table 3:** Serum urate (SU) change over 3-month periods in year 1 and flare occurrence at the next visit

Months	Flare assessment time	Flare status	*N*	Mean SU change (SD)	*p*	Patients with SU change > 30 µmol/L (%)	*p*-value, flare vs. no flare	Patients with SU change > 60 µmol/L (%)	*p*-value, flare vs. no flare	Patients with SU change > 90 µmol/L (%)	*p*-value, flare vs. no flare
0–3	Month 4	Flare +	41	170 (89)	0.13	95.1	0.63	85.4	0.33	78.0	0.86
	Month 4	Flare −	129	153 (85)		93.0		90.7		76.7	
3–6	Month 9	Flare +	30	19 (82)	0.42	36.7	0.71	30.0	0.66	16.7	0.56
	Month 9	Flare −	127	22 (71)		40.2		26.0		12.6	
6–9	Month 12	Flare +	47	10 (87)	0.36	38.3	0.43	31.3	0.55	14.9	0.24
	Month 12	Flare −	116	6 (67)		31.9		17.2		8.6	
9–12	Month 24	Flare +	41	− 1 (63)	0.15	14.6	0.043	7.3	0.25	7.3	0.67
	Month 24	Flare −	113	11 (63)		31.0		14.2		8.8	

Over 2 years time, no difference in flare was seen in those patients who, after being at target, again increased SU levels to > 360 mol/L or not, with a flare seen in 86.3% (88/102) among those with increased SU vs. 82.9% (68/82) in those without increased SU, *p* = 0.53. Corresponding findings were observed for patients with increased SU to > 400µmol/L with a flare in 81.0% (34/42) versus flare in 86.5% (122/141) among those without increased SU, *p* = 0.23 increase.

## Discussion

This study shows that fluctuation and change in SU are related to the occurrence of disease flare. Fluctuation (calculated as the sum of SU changes) was higher in patients with flares during year 1, but this was not observed for year 2. No relation to flares was seen for SU changes between adjacent visits, and not whether patients after initially reaching the treatment target again increased SU towards levels with increased risk of MSU crystal formation.

We found thus that the sum of absolute SU changes over an observation period was a useful indicator of fluctuation, being associated with the occurrence of flare during year 1. High fluctuation up to the 9-month visit was related to flares over 3-month periods, and fluctuation during months 0–3 was related to subsequent flares in months 3–6, thus indicating future flares.

Importantly, our study was performed in a clinical gout setting with intensive ULT, where SU was greatly reduced mainly in the first 3–4 months [17]. At best, a weak association between absolute SU values and flares has previously been reported [17, 18]. Acute gout flares can in the clinic be seen after ingestion of a purine-rich diet and are seen during ULT, possibly as a consequence of remodelling and mechanical disruption of the crystal aggregates with new exposure to monocytes [19], exposing a proinflammatory trigger [8]. In some conditions, SU fluctuation or change has been shown to play a role in increased flares, such as bariatric surgery [10] and pegloticase treatment which may lead to extensive SU fluctuations [9].

We studied a large patient cohort with frequent assessments of both SU and flares which allowed us to study their relationship. We acknowledge limitations in our study due to a one-centre design, lack of a control group excluding causal inferences, and that the study was performed in an intensive ULT setting with predominantly SU reductions. The study design did not allow for capturing rapid daily changes in serum urate due to diet or other factors.

In conclusion, we found that fluctuation in SU during 3-month periods and over the first year after the start with ULT was related to flares and even to subsequent flares in the first months. The sum of changes in SU was a useful way to measure fluctuation. To avoid fluctuations with possible flares, compliance with ULT treatment is important and colchicine prophylaxis over at least 6 months as recommended. Our findings require confirmation in other longitudinal studies. Findings support existing treatment recommendations with a focus on the gradual increase of ULT to lower SU [4, 5] and reinforce a need for treatment adherence to avoid SU fluctuation.

## Data Availability

The datasets generated during and/or analysed during the current study are available from the corresponding author upon reasonable request.
